# Sustainable Lipid Production with *Cutaneotrichosporon oleaginosus*: Insights into Metabolism, Feedstock Valorization and Bioprocess Development

**DOI:** 10.3390/microorganisms13091988

**Published:** 2025-08-26

**Authors:** Marion Ringel, Michael Paper, Marieke Willing, Max Schneider, Felix Melcher, Nikolaus I. Stellner, Thomas Brück

**Affiliations:** 1 Werner Siemens-Chair of Synthetic Biotechnology, TUM School of Natural Sciences, Technical University of Munich (TUM), Lichtenbergstraße 4, 85748 Garching, Germany; 2 TUMCREATE Ltd., 1 Create Way, Singapore 138602, Singapore

**Keywords:** *Cutaneotrichosporon oleaginosus*, microbial lipids, sustainable fermentation, carbon source alternatives, single-cell oil applications, single-cell oil production

## Abstract

The production of microbial lipids through single-cell oil (SCO) technologies has gained increasing attention as a sustainable alternative source of lipids for industrial applications. This development is driven by the limitations of plant-based oils, particularly their competition with food production and demand for arable land. *Cutaneotrichosporon oleaginosus* has been recognized as one of the most promising oleaginous microorganisms for efficient SCO production. To improve sustainability and economic viability, it is vital to understand the underlying metabolic mechanism of SCO production as well as needs and limitations in bioprocess engineering for the efficient utilization of carbon sources derived from diverse agricultural and industrial side streams. This review focuses on recent studies exploring the potential of SCO production through *C. oleaginosus* in a bioprocess context through the application of low-cost agro-industrial by-products as alternative carbon sources aiming to supply lipid raw materials for various industrial applications. *C. oleaginosus* can grow on different agro-industrial waste-derived substrates, including lignocellulosic biomass hydrolysates, biodiesel production process side streams, chitin-based by-products, cheese whey permeates, fungal biomass hydrolysates and algal biomass hydrolysates. These substrates contain various carbon sources, such as glucose, galactose, mannose, xylose, lactose, N-acetyl-glucosamine and glycerol, facilitating efficient SCO production. Additionally, the specific composition of SCO sourced from *C. oleaginosus*, including the presence of functional compounds like squalene and prevalent long-chain unsaturated fatty acids in its fatty acid profile, make it an ideal option to be used as a raw material in cosmetics, biofuel and food products. This comprehensive overview aims to shed light on the potential of *C. oleaginosus* in leveraging carbon source alternatives for sustainable SCO production for multifaceted, industrial applications of SCO.

## 1. Introduction

The 21st century has witnessed a notable surge in global population growth, leading to a corresponding rapid increase in global energy and food demands. More than 80% of these energy needs have historically been met by fossil fuels [1]. However, the depletion of fossil fuel reserves is imminent, prompting the exploration of alternative energy sources. Biofuels, derived from plant-based oils, have emerged as an alternative strategy to address the anticipated fossil fuel depletion and energy shortages [2]. Further, lipids for various industrial applications, including food applications, have traditionally been predominantly sourced from fruits or seeds of oleaginous plants. However, conventional agriculture for (oil) crop production often relies on monocultures and the use of pesticides and fertilizers with extensive land use to maximize space–time yields [3]. Traditional oil crops such as soy or oil palm have a particular negative impact on the environment due to unsustainable cultivation concepts which sacrifice rain forests for farmland to ensure a stable supply of food crops [4]. However, conventional approaches face significant challenges, including the effect of climate change, pests and crop diseases, as well as limited arable land, which hinder the efficient production of plant-based oils such as palm oil or cocoa butter [5,6,7]. These developments clearly highlight the importance of innovative, sustainable solutions for the pressing questions of food and energy security. In particular, the development of a more resource-independent, local and sustainable production of highly important platform chemicals, such as ‘oils and fats’, is needed to overcome the immanent challenges of climate change and surging need in global energy and food supply.

A promising alternative strategy to address these challenges is to harness circular bioeconomy approaches for the development of innovative production routes for, i.e., current fossil-based platform chemicals or fine oleochemicals [8,9]. Bioeconomic approaches often build on the development of biotechnological concepts which incorporate the utilization of renewable feedstocks, low-environmental-impact process designs and closing of the carbon cycle [10,11]. In this context, single-cell oil (SCO) produced by (oleaginous) microorganisms has gained attention in the recent past as a sustainable and scalable alternative to fossil and vegetable oils. SCO has a very diverse range of applications as its physico-chemical properties can be similar to fossil or vegetable oils, depending on the chemical composition of fatty acids. Thus, it may serve as a bio-based equivalent to conventional platform chemicals, opening the stage for SCO in applications such as nutraceuticals, edible oils and fats, cosmetics, and biofuels and lubricants, to name just a few [12,13,14]. Although it seems like a recent breakthrough in biotechnological research, the concept of SCO production utilizing microorganisms is far from new. Indeed, this process had already been in ‘industrial focus’ in Germany in the early stages of the 20th century during the turmoil of war, which led to shortages in conventional edible fat supplies [15]. Nevertheless, since bioprocess technology was still in its infancy, the early endeavours of SCO production were rather limited and thus never reached commercial exploitation [15]. In the following decades SCO production was very much focused on food and feed applications, but commercial exploitation was dependent on price volatility or shortages of certain specialty oils and fats [16]. For instance, a process for the production of a cocoa butter equivalent (CBE) was developed in the 1980s at a scale of 1000 tons per year using the oleaginous yeast *Cutaneotrichosporon oleaginosus*. The process was successfully scaled up to 250 m^3^ in a bubble column reactor, demonstrating its technical feasibility for industrial application and readiness for market deployment. However, with a drop in the price of cocoa butter and a price-competitive CBE made from palm oil readily available, the innovative project was dropped [16,17].

Oils and fats with designated use in the food and energy sectors are often bulk ingredients. Therefore, achieving a competitive price on the market, specifically with innovative, sustainable bioprocessing is a major factor for success. Specifically for biotechnological approaches, the road to cost efficiency is marked by three crucial pillars defining ‘make or break’ factors for an efficient bioprocess: (1) microbial strain selection, (2) media optimization in terms of process costs and productivity, and (3) efficient downstream processing [18]. The decision regarding a cost-effective media formulation is a crucial cost-driver in bioprocessing. In the context of a circular bioeconomy, this can, for instance, be achieved by using low-value biomass side streams that can be converted to a fermentable nutrient source [3,19].

This review aims to provide a comprehensive overview of recent advances and studies focusing on the utilization of novel industrial side streams as alternative substrates for cost-effective medium formulations for SCO production utilizing the oleaginous yeast *C. oleaginosus* as a microbial cell factory.

## 2. Far from Conventional—*Cutaneotrichosporon oleaginosus*, an SCO Production Powerhouse

En route to price-competitive bioprocessing, it is especially important to choose microbial production strains that are stable in their overall growth performance and production of the desired compounds. Further, to increase space–time yields, they should either thrive in a high-cell density fermentation setting or, despite lower cell densities, still be high-performance producers. Ideally the selected strain should also be very flexible regarding the nutrient source. This enables full utilization of the potential of a circular bioeconomy strategy by using very cost-effective but chemically diverse agrifood side streams [20]. Specifically in biotechnological production of SCO, oleaginous yeasts have been in the focus as potential industrial production hosts—not only for historical reasons. Generally, oleaginous microorganisms are capable of accumulating more than 20% of their dry cell weight (DCW) in lipids in the form of triacylglycerides (TAGs), which are typically referred to as single-cell oils [21,22,23,24,25,26]. Several yeasts have been identified for their potential in sustainable SCO production (Table 1), including, but not limited to, *Yarrowia lipolytica*, *Lipomyces starkey*, *Rhodosporidium toruloides*, *Rhodosporidium glutinis*, *Trichosporon fermentans* and *Cutaneotrichosporon oleaginosus* (previously known mostly as *Trichosporon oleaginosus* and also as *Cryptococcus curvatus*, *Apiotrichum curvatum* or *Candida curvata*).

Specifically in the context of scalable bioprocessing, oleaginous yeasts have the definite advantage of fast growth to high cell densities and high lipid contents of more than 60–70% [16,27]. Further, they show a remarkable substrate promiscuity which allows for the utilization of a broad range of biogenic waste streams [37,38]. In this context, *C. oleaginosus* stands out for its potential to utilize a particularly broad range of substrates and its high lipid accumulation capability, reaching levels of up to 85% of its DCW [27]. After several reclassifications throughout the years, *Cutaneotrichosporon oleaginosus* is currently classified as a basidiomycetous yeast within the domain Eukaryota, kingdom Fungi, phylum Basidiomycota, class Tremellomycetes, order Trichosporonales, family Trichosporonaceae, genus *Cutaneotrichosporon* [39]. Utilized substrates by *C. oleaginosus* encompass mono- and disaccharides, such as glucose, xylose, galactose, mannose, glucosamine, cellobiose and lactose, among others [40]. In this regard, no clear substrate preference has been found for different carbon sources; however, small differences in biomass production could be detected in shake-flask experiments: glucose, lactose, galactose, mannose, fructose, xylose, sucrose and maltose were found to be utilized rather efficiently. In the same setting, arabinose and sorbitol were proven to be less favourable carbon sources. [41]. In contrast to the broad tolerance of carbon sources, *C. oleaginosus* clearly shows a preference towards organic nitrogen sources such as yeast extract, tryptone/peptone and urea [41]. Interestingly, even more catabolically demanding substrates, such as lignin-derived aromatic compounds, raw glycerol derived from biodiesel production, alkanes from mineral oil refining and fatty acids from waste cooking oil, can also serve as nutrient source for *C. oleaginosus* [16,42,43]. It has to be noted that, while *C. oleaginosus* is generally very flexible towards different carbon sources, it shows varying biomass accumulation as well as differences in morphology on different substrates, indicating more demanding metabolic processes [37,40,41]. In this context, Shaigani et al. [37] and Awad et al. [41] conducted rather comprehensive studies on the influence of different carbon sources ranging from single sugars to complex hydrolysates on biomass production, lipid formation and fatty acid profile in shake-flask experiments. Both studies revealed that the choice of carbon source has an impact on the fatty acid profile. However, *C. oleaginosus* showed distinct differences in overall biomass and lipid yield for the different carbon sources studied. Thus, changes in its fatty acid profile might be influenced by drastic differences in intracellular lipid content. To this end, Rerop et al. [44] and Koruyucu et al. [28] cultivated *C. oleaginosus* in different controlled bioprocess settings in stirred-tank reactors using either consumption-based acetic acid supply with a coupled hydrolysate (lignocellulose or glucose/xylose/acetic acid model substrate) feed or phosphor-limiting conditions with glucose/mannose feed. Both studies revealed high lipid production but only marginal changes in the overall fatty acid profile. Therefore, the choice of carbon source seems to have a slight but not overarching effect on the fatty acid profile and needs to be evaluated for every SCO production process that should be set up in order to obtain the desired product.

Overall, the ability to grow on a wide range of agricultural and industrial side streams makes *C. oleaginosus* a very promising, efficient strain for cost-effective industrial-scale production of SCOs. Specifically, the usage of sugar-containing hydrolysates from industrial side streams for oleochemical production is a targeted approach to achieve the economic viability of sustainable SCO bioprocessing, thereby promoting bioeconomic circularity.

## 3. Understanding De Novo Lipid Formation—Key to Utilization of Biogenic Side Streams

**Figure 1:**
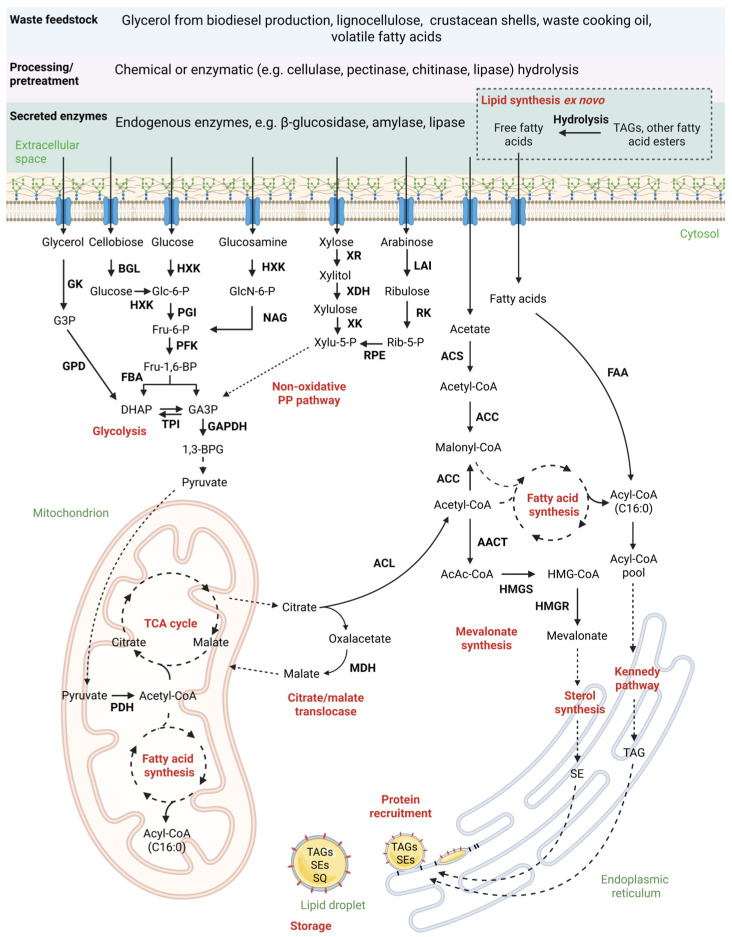
Metabolic pathways involved in fatty acid production by *C. oleaginosus*. The focus is on the generation of acetyl-CoA (Ac-CoA) as a central intermediate for fatty acid and isoprenoid production. Enzymes are highlighted in bold. The breakdown of the different amino acids and individual aromatic compounds as well as the phosphoketolase pathway were not considered. In the figure, AACT and HMGS are displayed in the cytosol, where they are most frequently found, but they have also been reported in other organelles such as the endoplasmic reticulum. The figure was adapted after [48,49,50,51,52,53,54,55,56]. 1,3-BPG: 1,3-Bisphosphoglycerate; AACT: Acetyl-CoA acetyltransferase; AcAc-CoA: Acetoacetyl-CoA; ACC: Acetyl CoA carboxylase; ACL: ATP citrate lyase; ACS: Acetyl-CoA synthetase; BGL: β-Glucosidase; DHAP: Dihydroxyacetone phosphate; FAA: Fatty acyl-CoA synthetase; FBA: Fructose-bisphosphate aldolase; Fru-1,6-BP: Fructose 1,6-bisphosphate; Fru-6-P: Fructose 6-phosphate; G3P: Glycerol 3-phosphate; GA3P: Glycerinaldehyde 3-phosphate; GAPDH: Glyceraldehyde 3-phosphate dehydrogenase; GK: Glycerol kinase; Glc-6-P: Glucose 6-phosphate; GlcN-6-P: N-Acetyl-glucosamine 6-phosphate; GPD: Glycerol-3-phosphate dehydrogenase; HMGR: Hydroxymethylglutaryl-coenzyme A reductase; HMGS: Hydroxymethylglutaryl-CoA synthase; HMG-CoA: Hydroxymethylglutaryl coenzyme A; HXK: Hexokinase; LAI: L-Arabinose isomerase; MDH: Malate dehydrogenase; NAG: N-Acetyl-β-d-glucosaminidase; PDH: Pyruvate dehydrogenase; PFK: Phosphofructokinase; PGI: Glucose-6-phosphate isomerase; PP: Pentose phosphate; RK: Ribokinase; Rib-5-P: Ribulose 5-phosphate; RPE: Ribulose 5-phosphate epimerase; SE: Sterol ester; SQ: Squalene; TAG: Triacylglyceride; TCA: Tricarboxylic acid; TPI: Triosephosphate isomerase; XDH: Xylitol dehydrogenase; Xylu-5-P: Xylulose 5-phosphate; XR: Xylose reductase. Created at https://BioRender.com.

To develop a bioprocess that efficiently utilizes biogenic side streams, it is instrumental to understand the underlying metabolic mechanisms. The de novo accumulation of lipids is a biological process which usually occurs as a stress response—typically during nutrient limitation (e.g., nitrogen or phosphorus) [45]. The accumulation of lipids then primarily serves as energy storage in the form of neutral lipids (e.g., TAGs). Conventionally, lipid accumulation is triggered through depletion of a nitrogen source. In a bioprocessing context, the carbon-to-nitrogen (C/N) ratio in the batch medium and feed input is a critical parameter that governs the potential for lipid accumulation. While the composition of the medium and feed can vary substantially between bioprocesses, particularly to accommodate the specific requirements of different species, C/N ratios below 20 are generally considered non-limiting, whereas ratios between 30 and 80 are associated with enhanced lipid production [45]. In oleaginous yeasts, the accumulation of lipids greatly relies on the availability of the universal fatty acid precursor acetyl-CoA (Ac-CoA) and the reduction equivalent NADPH [22,45,46]. Therefore, the involved metabolic steps can be divided into four distinct steps—(1) production of Ac-CoA; (2) biosynthesis of fatty acyl chains; (3) elongation, desaturation and TAG synthesis; and finally (4) lipid droplet biogenesis [45]. Ac-CoA, as a central metabolic intermediate, is supplied by upstream degradation of varying carbon sources. In most cases, this involves the degradation of hexose sugars via glycolysis with pyruvate as a key intermediate. Pyruvate is then transported to the mitochondria and subsequently converted to Ac-CoA [47]. However, other carbon sources, such as glycerol, disaccharides, organic acids or pentose sugars, can be used as substrates in this metabolic context. They are also metabolized to pyruvate as a general intermediate, but their utilization may involve different enzymatic pathways, such as phosphorylation of glycerol to glycerol-3-phosphate or uptake of xylose to xylose-5-phosphate via two additional enzymatic steps (Figure 1).

### 3.1. Step 1—Production of Ac-CoA

Conventionally, biotechnological lipid production is coupled with nutrient starvation. Depletion of, e.g., nitrogen in the batch medium leads to an activation of the adenosine monophosphate (AMP) deaminase, which decreases the cellular AMP content. As a result, isocitrate dehydrogenase (IDH) is downregulated within the mitochondria, leading to a significant reduction, or even a complete halt, of the citric acid cycle due to a decline in α-ketoglutarate levels. The cell now copes with accumulated isocitrate levels inside the mitochondria by upregulation of the aconitase to transform isocitrate into citrate, which is subsequently transported into the cytosol by the citrate/malate translocase [45,47]. Therefore, mitochondrial citrate levels are a major contributor to Ac-CoA available for lipogenesis under nitrogen limitation, which is underlined by the observation that oleaginous yeasts can show a three to four times higher intramitochondrial citrate level than non-oleaginous relatives [57]. In the cytosol, citrate is cleaved into acetate and oxalacetate. The latter is further converted into malate, which is then shuffled back into the mitochondrion (citrate/malate shuttle). Along this reaction route, reduction equivalents in the form of NADPH are built for lipogenesis. For further details, Garay et al. [45] give thorough insights into the generation of NADPH. However, it shall be noted that, particularly under nitrogen starvation, the enzymatic machinery for NADPH supply, more specifically malate dehydrogenase, has a significant influence on cellular upregulation of lipogenesis in oleaginous organisms [45]. In addition to the conventionally applied nitrogen starvation, it has been shown that the supply of intermediates of the fatty acid synthesis route as carbon sources can also lead to increased lipid accumulation while maintaining non-limiting growth conditions. An established example of this SCO production strategy is the metabolic shortcut taken in fatty acid synthesis by supplying acetic acid as a carbon source [58,59]. Under these conditions, acetic acid is imported into the cell and directly converted into Ac-CoA. It has been reported that supplying acetic acid as a carbon source *C. oleaginosus* induces lipogenesis without significant nutrient starvation [27,44].

### 3.2. Step 2—Biosynthesis of Fatty Acyl Chains

**Figure 2:**
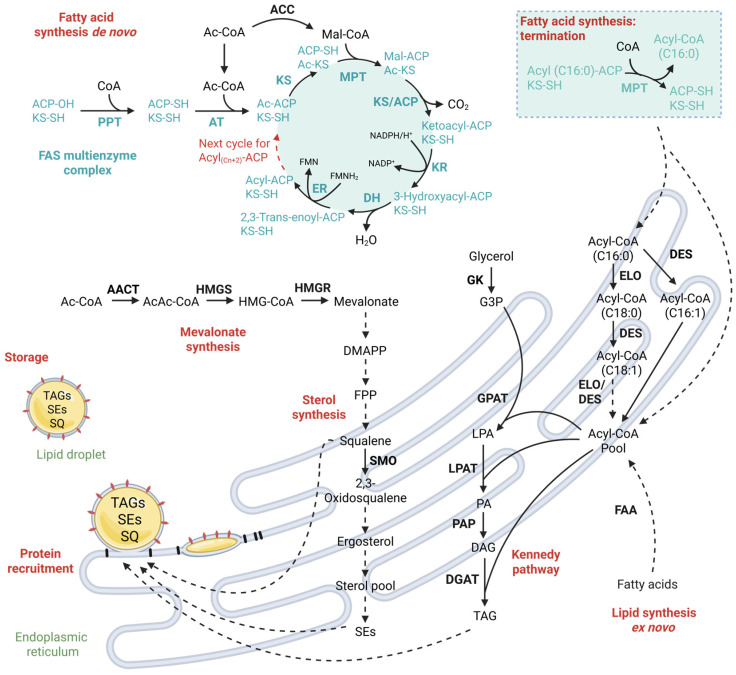
Metabolic pathways involved in fatty acid and isoprenoid synthesis in oleaginous yeasts. The enzymes of the fatty acid synthase are depicted in cyan colouring. Enzymes involved in the biosynthesis of sterols are present in yeasts in the cytosol, the endoplasmic reticulum, the peroxisomes as well as the LDs, but are displayed in the figure exclusively in the endoplasmic reticulum. The synthesis, elongation and desaturation of fatty acids is more strictly compartmentalized. A straight line indicates an enzyme reaction. A dotted line indicates a series of enzymatic reactions or other steps. Proteins recruited for lipid droplet formation are mainly perilipins (black) and seipin (red). The figure was adapted after [48,49,50,51,52]. AACT: Acetyl-CoA C-acetyltransferase; AcAc-CoA: Acetoacetyl-CoA; Ac-CoA: Acetyl-CoA; ACP: Acyl carrier protein; AT: Acetyltransferase; DAG: Diacylglycerol; DES: Desaturase; DGAT: Diacylglycerol acyltransferase; DH: Dehydratase; DMAPP: Dimethylallyl pyrophosphate; ELO: Elongase; ER: Enoyl reductase; FAA: Fatty acyl-CoA synthetase; FAS: Fatty acid synthase; FPP: Farnesyl pyrophosphate; G3P: Glycerol 3-phosphate; GK: Glycerol kinase; GPAT: Glycerol-3-phosphate acyltransferases; HMGR: Hydroxymethylglutaryl-coenzyme A reductase; HMGS: Hydroxymethylglutaryl-CoA synthase; HMG-CoA: Hydroxymethylglutarate-coenzyme A; KR: Ketoacyl reductase; KS: Ketoacyl synthase; LPA: Lysophosphatidic acid; LPAT: Lysophosphatidyl acyltransferase; MPT: Malonyl/palmitoyl transferase; PA: Phosphatidic acid; PAP: Phosphatidate phosphatase; PPT: Phosphopantetheinyl transferase; SE: Sterol ester; SMO: Squalene monooxygenase; SQ: Squalene; TAG: Triacylglyceride. Created at https://BioRender.com.

In the first step of de novo fatty acid synthesis, Ac-CoA is carboxylated by the addition of CO_2_ to malonyl-CoA (Mal-CoA) by acetyl-CoA carboxylase (ACC) [60]. In yeasts, ACC is encoded by the cytosolic ACC1, whereas HFA1 is present in mitochondria using biotin as a cofactor [45,60]. The fatty acyl chains are subsequently built from malonyl-CoA by the type I fatty acid synthase (FAS) enzyme machinery. In yeasts, the cytosolic type I FAS is composed of two distinct subunits organized in a α6β6 complex—FAS I (β-subunit) and FAS II (α-subunit) [60]. FAS I encompasses various enzyme functions, such as acetyl transferase, dehydratase, enoyl reductase, and malonyl and palmitoyl transferase activities [60,61]. FAS II includes acyl-carrier protein domain, 3-keto reductase, 3-keto synthase and phosphopantheteine transferase activities [60,62]. Other reviews, such as Schweizer et al.’s (2004) [63] and Tehlivets et al.’s (2007) [60], have discussed this aspect in great detail down to the exact molecular mechanisms. The reaction sequence from Ac-CoA towards fatty acyl chains follows a distinct pathway in yeasts (Figure 2). Firstly, Ac-CoA is loaded to the 3-ketoacyl synthase (KS) with the help of an acyl-carrier protein (ACP) which temporarily holds the Ac-CoA. KS then catalyzes the fusion of Ac-CoA with Mal-CoA to form 3-ketoacyl-ACP/CoA. The newly formed molecule holding a ketoacyl-group is subsequently reduced by the 3-ketoacyl reductase (KR) to 3-hydroxyacyl-ACP/CoA. An enoyl dehydratase (DH) abstracts water to form 2,3-trans-enoyl-ACP/CoA, which is finally reduced to acyl_(Cn+2)_-ACP/CoA by enoyl reductase (ER). This reaction pathway is repeated until a 16-carbon fatty acyl chain (as palmitoyl-ACP) is formed (Figure 2). Yeasts subsequently employ a malonyl/palmitoyl transferase (MPT) to transfer the acyl chain from ACP to CoA [45,60,61,63].

### 3.3. Step 3—Elongation, Desaturation and TAG Synthesis

Next, palmitoyl-CoA is channelled to the endoplasmic reticulum (ER), where it is elongated to up to C26 by elongases, following a similar reaction pattern as in FAS I [45]. Beyond this, acyl chains can be dehydrogenated by desaturases. Most commonly, the Δ9 position, but also Δ12 or Δ15, are subject to desaturation, thereby creating fatty acids comprising C18:1, C18:2 or C18:3 [64]. From this fatty acid pool (in the form of an acyl-CoA), some entities are designated for use in membranes or for other cell functions, or they need to be stored effectively in neutral lipids. In oleaginous yeasts, fatty acids are mainly stored in the form of TAGs. The main precursors for TAGs, phosphatidic acid (PA) and diacylglyceride (DAG), are yielded from two major de novo pathways, either originating from glycerol-3-phosphate (G-3-P) or dihydroxyacetone-phosphate (DHAP). G-3-P is acylated by G-3-P acyltransferase (GPAT) at the *sn-1* position to form 1-acyl-G-3-P (lyso-phosphatic-acid, LPA) and then at the *sn-2* position to form PA by the 1-acyl-G-3-P-acyltransferase (LPAT) (Figure 2). Alternatively, DHAP is acylated at the *sn-1* position by DHAP acyltransferase; subsequently reduced by 1-acyl-DHAP reductase, yielding LPA; and then further acylated by AGAT to yield PA. At this stage, PA can also form phospholipids for membrane biosynthesis. A subsequent dephosphorylation of PA by phosphatidate phosphatase (PAP) yields DAG, which can then be further acylated by diacylglycerol acyltransferases (DGATs) to obtain TAGs [65]. At this stage, we would like to highlight the very detailed work on triacylglycerol biosynthesis in yeast by Sorger et. al. [65].

### 3.4. Step 4—Lipid Droplet Biogenesis

Finally, accumulated TAGs need to be stored safely inside the cell. To this end, lipid droplet (LD) formation has evolved in cells to store excessively produced neutral lipids enclosed by a phospholipid monolayer. Additionally, LDs can store other neutral lipids such as squalene, sterol esters (SEs from different intermediates of sterol biosynthesis) and even fat-soluble secondary metabolites (e.g., terpenes and vitamins) (Figure 2) [66]. Recent research on the biosynthesis of LDs has shown that TAG and LD biosynthesis takes place between the two membrane leaflets of the ER [45,67]. TAGs begin to accumulate within the ER membrane and generate a lens-like protrusion [45,68]. PAT proteins (short for perilipin, adipocyte differentiation-related protein and TIP47) subsequently enclose the newly formed lens from the outer membrane layer [45,68]. Once the LD has accumulated enough TAGs, the outer membrane buds off and the LD is formed [45]. Although LDs are primarily storage organelles for neutral lipids, it has been reported that proteins with enzymatic functions in lipid metabolism or other proteins of structural importance are also associated with LDs. This highlights that LDs are contributing to a highly complex metabolic network sustaining lipid homeostasis [67,69,70].

## 4. Ex Novo Lipid Synthesis—Harnessing the Potential of Oily Waste Streams

De novo lipid biosynthesis uses conventional sugars or short-chain organic acids as carbon sources, which are metabolized through the key intermediates pyruvate and Ac-CoA. Oleaginous yeasts are also capable of assimilating hydrophobic substances such as waste cooking oil to form lipids ex novo. In a study comparing the growth and lipid accumulation performance of conventional glucose and waste cooking oil as carbon sources, *C. oleaginosus* demonstrated considerable potential for utilizing oils as an alternative carbon source. [56]. Other oleaginous yeasts capable of hydrophobic substance utilization are, for instance, *Yarrowia lipolytica*, *Cryptococcus* sp., *Rhodosporidium* sp., *Geotrichum* sp. and *Trichosporon* sp. [71]. Fatty acids and TAGs are taken up with active transporters or even simple diffusion. TAGs are hydrolyzed to their respective free fatty acids and activated with CoA to form acyl-CoA [56,71]. The created acyl-CoA is channelled into the cytosolic acyl-CoA pool and may be further processed as described (Figure 1).

## 5. Bioprocessing for SCO Production

Achieving cost-effective production of single-cell oils (SCOs) remains a central objective in developing competitive biotechnological alternatives to conventional lipid sources. A key benchmark for commercial viability is reaching cost parity with established market products. In this context, there are several cost drivers that need to be taken into consideration when developing an SCO production process. Next to the strain selection, the commercial viability of an SCO production process is dependent on process engineering factors such as feedstock costs, bioprocessing equipment, process design and downstream processing efficiency [72,73]. Naturally, the capital expenditure (CAPEX), including the cost of bioreactors, auxiliary equipment and facility infrastructure, alongside operational expenditure (OPEX), including energy, maintenance and labour, collectively contribute to overall production costs. Since CAPEX-related investments are largely fixed and essential for facility upkeep, the greatest potential for cost reduction lies in optimizing operational aspects, including raw material selection and bioprocess design [74]. A closer examination of bioprocess equipment and process design is therefore warranted. Biotechnological SCO production using oleaginous yeast, specifically *C. oleaginosus*, requires a standard, stirred-tank bioreactor design for an aerobic fermentation set-up with various controlled parameters such as pH, temperature and dissolved oxygen content. To this end, optimal growth is generally observed for *C. oleaginosus* at 28–30 °C and pH 5.5–6.5, with aerobic conditions maintained at dissolved oxygen levels above 30% to support both cell proliferation and lipid biosynthesis [41,75]. Lipid production is usually triggered through nutrient limitation, e.g., phosphate or nitrogen [28,76,77]. Additional strategies for lipid formation may include supply of key intermediates of fatty acid synthesis. For example, the combination of continuous acetic acid supply and other carbon sources, such as carbohydrate-rich hydrolysates, yields high lipid contents in *C. oleaginosus*.

Further, the exact bioreactor design must adapt to the requirements and limitations of the used microorganism and the complete process. Factors to consider include oxygen demand, shear sensitivity, sterilization requirements for aseptic processing, foaming behaviour, production scale, and the number and type of seed reactors or media tanks required [78,79]. Detailed elaboration of the engineering of bioreactors for SCO production is thus a highly complex topic and beyond the scope of this review. Therefore, we would like to point towards the work of Mersmann [78] and Storhas [80] for detailed elaboration of bioreactor design and operation. Nevertheless, from an economic perspective, selecting the most appropriate equipment and bioreactor operation mode is fundamental to process efficiency.

Specifically, the bioreactor operation mode can contribute significantly to the overall costs of a bioprocess. Generally, bioreactors can be operated in batch/fed-batch or continuous mode. Batch/fed-batch processes typically excel in small-batch bioprocesses with expensive products, while continuous mode is recommended for high-capacity plants for the production of low-value products [78]. To this end, *C. oleaginosus* has been commonly cultivated in fed-batch cultivation strategies [27,28,44]. However, inevitable advantages of continuous mode are, i.e., low investment cost, better heat recovery, low energy cost for sterilization, low labour cost, better process control and the possibility of better adjustment to continuous downstream processes and process-integrated removal of the bioproduct if possible [78]. Although these are advantages that specifically contribute to cost-effectiveness, there might be reasons against developing a continuous bioprocess, such as genetic instability of the microorganism, contamination of the process or resulting difficulties in downstream processing [78]. *C. oleaginosus* would generally be suitable for cultivation in continuous mode. However, efficient downstream processing of the high-lipid-content yeast cells largely relies on reaching a sufficient lipid content inside the single cells in order to efficiently separate the lipid and water phases. Therefore, setting up a continuous operation mode in the fermentation needs to be perfectly adapted to the needs of downstream processing in order to avoid differences in cell population concerning the lipid content. To the best of our knowledge, however, further research initiatives are needed to set up a well-adapted continuous-mode bioprocess for *C. oleaginosus*. In conclusion, the choice of a bioreactor or process design for SCO production needs to adapt to the process needs and might after all be a compromise of cost versus process efficiency.

## 6. Enhancing *C. oleaginosus* SCO Bioprocessing Through Genetic Engineering Approaches

Although recent approaches in bioprocess optimization using wild-type *C. oleaginosus* were rather successful, reaching up to 85% *w*/*w* in lipid content, genetic engineering is a promising strategy to further improve the lipid yield or introduce targeted changes in the fatty acid profile [27,44]. Specifically, the latter might allow for the production of tailored fats and oils solutions in, e.g., the food or cosmetic market. Nevertheless, it needs to be added here that products that originate from genetically modified organisms have to face harsh regulatory hurdles before being allowed on the market. Putting this challenge aside, approaches like random mutagenesis using N-Methyl-N′-nitro-N′-nitrosoguanidine (MNNG) and acridine mustard (ICR-170) have proven to be successful for *C. oleaginosus* [81]. These approaches yielded mutants with altered fatty acid profiles [82]. However, random mutagenesis is certainly not the preferred choice when aiming for targeted adaption of a production host. Other strategies comprise agrobacterium-mediated transformation [82,83], CRISPR/Cas-based targeted editing [84] and model-driven metabolic engineering via co-transformation of plasmids [85]. We refrain from a more detailed elaboration of the different methods here. However, we would like to highlight that, specifically for targeted engineering of the fatty acid profile but also for elevating lipid yield, genetic engineering is a promising approach. Shaigani et al. achieved a 54% boost in total lipid titre in a genetically engineered strain overexpressing a Δ9-desaturase [84]. Koivuranta et al. successfully elevated lipid yields in non-limiting conditions by overexpression of a pyruvate dehydrogenase using glucose and xylose as carbon sources [86]. Further, Duman-Özdamar et al. were able to increase the lipid yield by 1.4-fold through overexpression of key enzymes such as ATP-citrate lyase, acetyl-CoA carboxylase, threonine synthase and hydroxymethylglutaryl-CoA synthase using genetic engineering techniques [85]. Other approaches for genetic engineering of SCO production involve strategies for flocculation of cells for easier harvesting by overexpression of adhesin cell flocculin 1 [87]. All the mentioned engineering approaches are included in Table 2 for a better overview of goals, genes of interest and used transformation methods. Generally, while there are already promising approaches for genetically optimizing *C. oleaginosus* for (tailored) SCO production, there is still a large open space for future scientific endeavours.

## 7. Industrial Waste as an Alternative Substrate for Oil Production with *C. oleaginosus*

**Figure 3:**
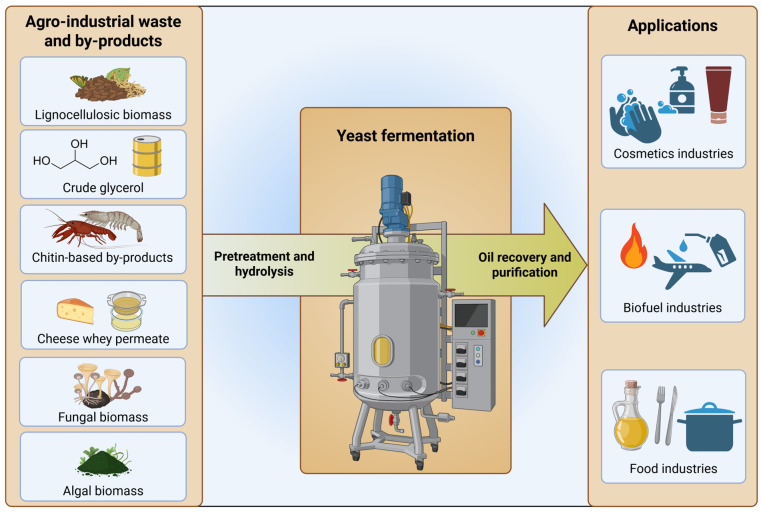
Agro-industrial waste and by-products for *C. oleaginosus* fermentation to produce SCO and the applications of the produced lipids. Created at https://BioRender.com.

Further, apart from the process engineering aspect, substrates, such as the carbon source used in the process, contribute largely to the end product’s price tag [89,90]. Specifically, oils and fats used in the food or energy sector are considered bulk chemicals and thus need to be at a competitive price compared to traditional oil and fat sources. In this context, biogenic side streams from (agro-)industrial sources are very attractive and sustainable nutrient sources for bioconversion into higher value products (Figure 3). Biogenic feedstocks, such as corn or sugarcane juice, have even already been successfully applied in first-generation biofuel production, thereby highlighting the potential for setting up a sustainable, cost-effective biorefinery [89].

The economic feasibility of SCO production can thus be supported by utilizing low-value biogenic side streams as raw materials derived from agro-industrial wastes, by-products and biomasses. The exploration and upcycling of diverse side streams not only contribute to the economic viability of the process but also align with the broader goal of creating a more sustainable and circular bio-economy. To this end, all carbon sources containing carbohydrates (hexoses or pentoses) with negative or low market values and some minerals and trace elements can be considered as potential substrates [91]. Several biogenic side streams have already been tested for their suitability as feedstocks for *C. oleaginosus*. These comprise, e.g., by-products of the biodiesel production process (crude glycerol), corn and corn stover hydrolysate, chitin-based by-products (N-acetylglucosamine), plant-based (lignocellulosic) biomass hydrolysates, cellulosic wastepaper hydrolysates, cheese whey permeate, herbal extraction residue hydrolysate, microalgae biomass hydrolysate, and other by-products (Table 3) [49,91,92,93].

In this context, the composition of biogenic side streams as potential substrates for fermentation is crucial information for tuning the medium composition for an efficient lipid production. Biogenic waste streams usually consist of any form of polymeric sugar such as cellulose or glucan. For efficient conversion of biomass to SCO, the feedstocks typically need to undergo a pretreatment to mobilize the polymeric sugars. The exact composition of the polymers as well as the additional components vary from biomass to biomass. Thus, the pretreatment needs to be adapted for each new biomass, and it certainly needs to be able to compensate for small changes in the raw materials. Ideal biomass pretreatments should avoid energy-intensive steps (e.g., heating), avoid formation of inhibitory compounds and maximize sugar release for efficient conversion into SCO [89]. To this end, it should be noted that some organic compounds, such as furfural and 5-hydroxymethylfurfural, can reduce yeast growth and lipid production [99]. Accordingly, to improve cell biomass and lipid production, the biomass pretreatment for nutrient-rich hydrolysates must be adapted to the microorganisms used, depending on their susceptibility to inhibitory compounds, and in the best case the formation of inhibitory compounds must be avoided [94,100]. Pretreatments could involve chemical (acidic or basic hydrolysis) or physical methods (e.g., heat treatment or steam explosion) or enzymatic hydrolysis [101]. Enzymatic treatment of biomass has proven to be a very promising approach for sugar mobilization, as it operates at low temperatures, thereby avoiding high energy costs and unwanted side products from heat treatment [102,103].

To date, several biogenic side streams have already been evaluated as substrates for *C. oleaginosus* SCO production due to their wide tolerance to different carbon sources. The specific composition as well as performance as feedstock of some should be highlighted in the following [16,91].

### 7.1. Lignocellulosic Biomass

Lignocellulosic biomasses (LBs), such as grass, wood and agricultural residues, are promising and valuable by-products derived from agricultural conversion and processing industries. LB is mainly composed of cellulose, hemicellulose and lignin. Cellulose, the most abundant polymer in LB, constitutes 40–60% of the dry weight [104]. It is composed of glucose monosaccharides linked via β-1,4-glycosidc bonds with a degree of polymerization in the range of 150–5500 hexose subunits and typically around 7000–15,000 glucose monomers per cellulose polysaccharide [104]. Hemicelluloses are heteropolysaccharides containing different hexose subunits (e.g., glucose, galactose and mannose), pentose sugars (e.g., xylose and arabinose) and sugar acids (e.g., glucuronic acid and galacturonic acid) [104]. Hemicelluloses have a lower degree of polymerization (50–200) compared to cellulose and a shorter chain length (500–300 sugar monomers). Approximately 20–35% of LB is composed of hemicelluloses [105]. While celluloses have a rather defined composition, hemicelluloses can vary significantly and are grouped as hemicellulosic sugars. These comprise, e.g., xylans (hemicellulose from 1,4-linked β-D-xylose monomers) and mannans (mannose monomers β-1,4-linked). Mannans vary in their composition and also include, though they are not limited to, glucomannan (glucose–mannose), galactomannan (galactose–mannose), glucuronic acid and galacturonic acid [104]. The exact composition of hemicellulosic polysaccharides largely depends on their sources, e.g., xylan from birch wood contains 89.3 wt% xylose, 1.4 wt% glucose, 1 wt% arabinose and 8.3 wt% anhydrouronic acid [104,106]. In this regard, we would like to point to the work of Okolie et al. [104], who give a very comprehensive insight into the composition of lignocellulosic biomass. Cellulose and hemicelluloses can be easily enzymatically hydrolyzed and used as fermentation feedstocks. Lignin, as the second most abundant biopolymer in LB (15–40%), provides a protective role for these homo- and heteropolysaccharides against microbial attacks [105,107,108]. It is randomly assembled from phenylpropane monomers, and its basic building blocks include p-coumaryl alcohol, coniferyl alcohol and sinapyl alcohol [104]. Based on the source of biomass, LBs are categorized into different types, including forestry waste, energy crops, agricultural residues, animal manure and lignocellulosic industrial wastes. These resources differ in the content and structure of the main LB compositions (cellulose, hemicellulose and lignin). Since LB provides an appropriate amount of carbohydrates with a sufficient amount of proteins, lipids, trace elements and other nutrients, LB is a suitable and applicable substrate for industrial-scale fermentation processes [101,109]. The close associations formed among cellulose, hemicellulose and lignin polymers in LBs, facilitated by covalent linkages and hydrogen bonds, necessitate specific chemical and biological dissociation and depolymerization processes to convert the starting LB into a suitable fermentative substrate [107,109]. Thermochemical strategies involving chemical pretreatment with different acids, bases and solvents, as well as thermal processing using steam explosion or microwave irradiation, are typically required. Optimization of these treatment approaches is crucial to minimize sugar and nutrient degradation [101,110].

LBs have also been used as substrates for *C. oleaginosus* fermentation and lipid production [23,37,84,111]. For instance, Caporusso et al. (2021) successfully employed and developed cardoon-stalk hydrolysates as a substrate for batch-mode *C. oleaginosus* fermentation [94]. They observed the formation of significant levels of toxic compounds, including furfural and hydroxymethylfurfural, during the steam explosion treatment of the cardoon-stalk hydrolysates. These compounds required detoxification before fermentation. During batch fermentation, the researchers achieved a maximum lipid yield of 7.1 g/L [94]. Rerop et al. (2023) developed an acetic acid-based fed-batch fermentation process using *C. oleaginosus* for the transformation of a pentose-rich LB hydrolysate, sourced from an industrial paper mill, into SCO [44]. They found that pentose sugars as a carbon source and an alternative for glucose in this substrate significantly improved SCO production during the fermentation process. Simultaneous uptake and consumption of xylose and glucose were reported, with a maximum lipid yield of 42.1 g/L. Since most of the lignocellulose-based industrial waste is pentose-rich, this could be considered as a valuable and sustainable substrate for industrial fermentations using *C. oleaginosus*.

Agricultural by-products, such as residues from herbal extraction and hydrolysates from corn stover, were also used as LB-based substrates for *C. oleaginosus* fermentation and SCO production. In the study of Wang et al. (2022), corn stover was enzymatically hydrolyzed during fed-batch *C. oleaginosus* fermentation for SCO and biodiesel production [95]. These researchers achieved a maximum lipid yield of 42.3 g/L. The fatty acid profile of the produced oil was also found to be suitable for high-quality fuel production [91,95]. Additionally, Zhang et al. (2022) evaluated hydrolyzed herbal extract residues as the main substrates for *C. oleaginosus* batch fermentation to produce biodiesel [96]. They treated the herbal extract residues with diluted sulfuric acid before fed-batch enzymatic hydrolysis, achieving high oleaginicity with 8.5 g/L and 40.7% lipid concentration and content, respectively [96].

### 7.2. By-Products of Biodiesel Production (Crude Glycerol)

Production of biodiesel, which has been considered one of the renewable and sustainable alternatives for fossil fuels, has recently increased. A significant by-product of this process is crude glycerol, accounting for approximately 10% (*w*/*w*) of the total by-products generated during biodiesel production. Globally, it is estimated that over 3.7 billion gallons of crude glycerol are produced annually [111,112,113,114]. Crude glycerol is of high value, and several applications have been developed to valorize this commercial by-product. Since the production cost of biodiesel is comparatively high, extensive research has focused on the direct utilization of glycerol in chemical and biological industries [115,116]. Since oleaginous yeasts such as *C. oleaginosus* or *Schizochytrium limacinum* are able to utilize crude glycerol as their primary carbon source for lipid production, this compound has been used as a substrate for SCO production. However, high concentrations of glycerol (>10%) can exert toxic effects on the cell growth rate of oleaginous yeasts. Therefore, optimal concentrations of glycerol are required for efficient yeast fermentation and SCO production [116,117]. Pham et al. (2021) demonstrated that *C. oleaginosus* is able to utilize crude glycerol waste as a carbon source for SCO production without generating toxic compounds during the fermentation [49]. This yeast can accumulate more than 50% (*w*/*w*) of intracellular lipids under these conditions. *C. oleaginosus* has been found to produce significantly higher lipid yields compared to other oleaginous yeasts and lipid-producing microalgae when using glycerol as a carbon source. Furthermore, the fatty acid profiles of the lipids produced by *C. oleaginosus* grown on crude glycerol contain comparatively higher concentrations of monounsaturated fatty acids such as oleic acid (C18:1), which is favourable for biofuel production [49,117].

### 7.3. Chitin-Based By-Products

Chitin is the second most abundant biopolymer after cellulose and is based on β-1,4 N-acetyl-glucosamine (NAGA). It can be found in insects (insect epidermises), most fungi (fungal cell walls) and crustaceans (shrimp shells). Each year, more than 100 billion tons of chitin-rich materials (squid, shrimp shells, insect epidermises, etc.) are produced globally in marine and seafood industries [118,119,120]. Crustaceans like crabs and shrimp contain 15–40% (*w*/*w* dry weight) chitin. Further, around 15% (*w*/*w* dry weight) of the fungal cell wall is made of chitin. The chitin content of insect epidermises ranges from 20 to 40% (*w*/*w* dry weight). However, the majority of chitin-rich waste and by-products are discharged into nature, thus contributing to environmental pollution and leaving this valuable source of feed stock scarcely exploited [119,121]. In this regard, selected oleaginous yeasts such as *C. oleaginosus*, *Trichosporon cutaneum*, *Cryptococcus albidus* and *Trichosporon fementans* are able to directly utilize chitin as a carbon source for lipid production. Generally, for biological degradation of chitin, chitinase enzymatic activity is required. Many of the oleaginous yeasts have been identified to produce different types of extracellular chitinases. Specifically, Tang et al. (2020) demonstrated the efficient conversion of chitin-based materials into SCO by *C. oleaginosus* [97]. Shaigani et al. (2021) showed that *C. oleaginosus* is capable to metabolize up to 66% (*w*/*w*) of available NAGA as a carbon source during fermentation, resulting in lipid production with elevated levels of unsaturated fatty acids (C18:2) [37]. Fatty acid compositional profiles of SCO produced by oleaginous yeast fermentation on chitin-based substrates are comparable with those from fermentation on other substrates. These lipid profiles were mainly composed of oleic acid, palmitic acid, stearic acid and linoleic acid (Table 4) [37,91,97].

### 7.4. Cheese Whey Permeate

Cheese whey permeate (CWP) is a high-value by-product of dairy industries containing protein (0.6–0.8%), lactose (4–5%), lipids (0.4–0.5%), peptides, various micronutrients and different minerals (55% of milk nutrients). It is generated during the cheese production and coagulation process of milk casein and is obtained through ultrafiltration of cheese whey [122,123]. Approximately 10 L of CWP is produced after the production of 1 kg of cheese. It is estimated that more than 100 million tons of CWP are generated globally each year. Different cheese whey-derived products, such as dried whey, concentrated whey and whey hydrolysate, have been used as substrates and raw materials for the production of a wide range of biological products, including lactic acid, biomass, ethanol and biofuel. The fermentation of cheese whey by-products to produce SCO and single-cell protein has been suggested, developed, optimized and successfully evaluated by several researchers [124,125]. Donzella et al. (2022) developed and optimized the bioprocess for valorization of liquid CWP via *C. oleaginosus* two-step fermentation to produce SCO [98]. They reported a high fermentation yield with a final lipid concentration of 38 g/L [98]. Notably, some oleaginous yeast strains, such as specific species of *Geotrichum* and *Galactomyces* yeasts, were originally isolated from dairy farms and industry equipment [126,127].

### 7.5. Fungal Biomass

Fungal biomasses are carbohydrate-rich, low-value carbon sources which have recently been identified as potential substrate alternatives for industrial fermentation. In addition to different hexoses and pentoses, these by-products contain various minerals and trace elements required for a wide range of microbial fermentations [128,129]. Approximately 50–80% of the dry weight of the fungal biomasses is composed of carbohydrates. The amounts of proteins, lipids, chitin constituents and minerals are in the ranges of 3–20%, 1–10%, 1–20% and 1–5% of the dry weight of fungal biomasses, respectively [130,131]. Considering the valuable composition of fungal biomasses, hydrolysates of these by-products have the potential to be used as feedstocks for *C. oleaginosus* and other microbial lipid producers [132,133].

*Aspergillus niger* is a filamentous fungus, known for its industrial use in citric acid production [134]. The biomass derived from *A. niger* is composed of different hexoses (glucose, galactose and mannose), chitin, glucosamine, proteins, β-glucans and a wide range of trace elements. The composition of *A. niger* biomass and the large-scale global production of this by-product makes it an ideal potential substrate alternative for *C. oleaginosus* fermentation in the production of SCO [135,136,137,138].

### 7.6. Algal and Microalgal Biomass

Algae, especially microalgae, exhibit superior efficiency in carbon dioxide fixation through photosynthesis compared to traditional land crops. In addition, their rate of biomass formation, as well as their areal oil production rate, are significantly higher [139,140]. Moreover, waste, brackish or sea water can be used for the cultivation of marine microalgae, thereby avoiding competition with freshwater resources [141,142,143]. Although SCO production using microalgae can be more efficient than using traditional crops, it is often induced by nutrient limitation, resulting in reduced growth and increased carbon dioxide consumption compared to optimal conditions. A potential solution lies in a two-step process that combines the efficient carbon capture properties of microalgae with effective lipid production using oleaginous yeasts. In this approach, microalgae are cultivated under optimal conditions for carbon capture, and the resulting microalgae biomass is subsequently hydrolyzed. The resulting sugar-rich hydrolysate is further used for SCO production by oleaginous yeasts [144]. For instance, Younes et al. (2020) used the hydrolysates of microalgae biomass as substrates for *C. oleaginosus* and microbial lipid production [92]. The researchers cultivated *C. oleaginosus* and other oleaginous yeasts on biomass of *Scenedesmus obtusiusculus*, which was hydrolyzed in a single-step process. They noted that *C. oleaginosus* is able to utilize microalgae cell-based residues as the main substrates for yeast lipid production. This resulted in *C. oleaginosus* cultivation yields of 3.6 g/L and 35% for lipid concentration and lipid content, respectively [92]. Furthermore, Meo et al. (2017) used a *Scenedesmus* hydrolysate with phosphate precipitation in a membrane bioreactor for lipid production using *C. oleaginosus* [76]. Their approach yielded a biomass concentration of 58 g/L with a lipid content of 53% (*w*/*w*) [76]. Koruyucu et al. even outperformed these results with a similar approach using a membrane bioreactor operated in semi-continuous mode, thereby achieving 116 g/L biomass concentration with an intracellular lipid content of up to 76.5% (*w*/*w*) [28].

## 8. Industrial Applications of SCO Produced by *C. oleaginosus* Fermentation

SCO production using low-cost biogenic side streams presents a promising pathway toward improved sustainability in various industrial sectors. Due to their similar physico-chemical properties to conventional plant-derived oils and fats, SCOs can serve as drop-in alternatives, enabling their seamless integration into existing production pipelines without the need for extensive process modifications [145]. In this context, SCOs exhibit considerable versatility as raw materials across various industries, such as human health, cosmetics, food production, animal feed production, biopolymer production and bioenergy. Therefore, the sustainable biosynthesis of SCO has become one of the most vibrant and fastest-growing research topics in the fields of biotechnology and industrial microbiology in recent years, with ever-growing attention from industrial stakeholders [146,147]. Chemically, SCOs are sources of glycerol, free fatty acids, fatty alcohols and fatty acid methyl esters, which can be subsequently converted into other useful products, such as soaps, fatty amines and other lipid-based derivative products. These products provide a broad range of applications, such as production of bio-surfactants, waxes, paints, lubricants and biofuels [16,27,91,147].

Biofuel production from *C. oleaginosus* SCO is definitely a feasible option due to its chemical composition. Its lipid profile is well suited for conventional biodiesel processes, which makes it a feasible drop-in replacement for petroleum-derived diesel, or it may be used as an additive for sustainable aviation fuel [16,92,93,117]. Nonetheless, achieving economic competitiveness with fossil diesel remains a significant challenge.

Beyond bulk biofuels, *C. oleaginosus*-derived SCOs offer potential for higher-value applications. SCO from *C. oleaginosus* was shown to have a comparably high content of commercially relevant secondary compounds such as squalene and ergosterol. Specifically, squalene is a non-toxic lipophilic hydrocarbon that has been widely used as an antioxidant, emollient and moisture-retaining agent in cosmetics industries, especially in the formulation of skincare products. Traditionally sourced from deep-see shark liver oil, squalene has been both expensive and environmentally problematic [148]. Microbial production via *C. oleaginosus* offers a more ethical, scalable and sustainable alternative. Stellner et al. (2023) optimized and enhanced the squalene production capacity of *C. oleaginosus*, achieving an impressive cellular accumulation of up to 2169 mg/100 g of the derived SCO [148]. This was achieved by employing the squalene monooxygenase inhibitor terbinafine during a fed-batch fermentation process, showcasing the potential of *C. oleaginosus* as a microbial cell factory for the production of high-value compounds [148].

Further, the cosmetic industry, as well as the food industry, has shown growing interest in finding sustainable alternatives to palm oil in their formulations. Yeast lipid-derived edible oil can extend to various applications, functioning as a palm oil substitute or as a cocoa butter equivalent [16]. Palm oil, which is mainly composed of palmitic acid (44%), is predominantly produced in tropical environments of Asia and South America, with an annual global production over 80 million tons [149]. Due to their SCOs’ compositional similarity to palm oil, certain strains of oleaginous yeasts, such as *C. oleaginosus*, *L. starkeyi* and *R. glutinis*, were used to produce palm oil substitutes with comparable functional qualities [4,150,151]. Beyond palm oil alternatives, yeast oil SCOs produced by *C. oleaginosus*, *S. cerevisiae* and *Y. lipolytica* have been developed and used as cocoa butter equivalents [16,152,153]. Cocoa butter, extracted from cocoa beans, is predominantly composed of saturated fatty acids (approx. 60%) and has a distinct cocoa flavour. Due to its particular fatty acid profile, cocoa butter is solid at room temperature and immediately melts inside the mouth contributing to a specific sensory experience [154]. Cocoa butter equivalents must have a similar fatty acid profile to be able to mimic these technical sensory properties. Cocoa butter is relatively expensive and serves as a key raw material in several food industries. Consequently, sustainable and affordable oil substitutes derived from other plants or SCOs with similar fatty acid compositions and physico-rheological properties are of great interest [155,156].

## 9. Conclusions

*C. oleaginosus* has emerged as one of the most promising candidates among oleaginous yeasts for the sustainable conversion of a wide range of agricultural and industrial wastes and by-products to SCO. This yeast’s robust metabolic flexibility and high lipid accumulation capacity make it particularly valuable for circular bioeconomy models. An improved understanding of the underlying lipid metabolism in *C. oleaginosus* has highlighted key metabolic nodes such as the citrate level in mitochondria, the cellular Ac-CoA supply route, NADPH-generating pathways and regulatory elements governing lipid accumulation under nutrient-limited conditions. These aspects provide potential leverage points for further optimizing the bioprocess of SCO production and achieving higher lipid yields. Various recent studies have demonstrated that *C. oleaginosus* is able to grow fast and utilize different carbon sources, such as monosaccharides (glucose, galactose, mannose, xylose, etc.), disaccharides (lactose), N-acetyl-glucosamine and glycerol. These substrates can be derived from a variety of agro-industrial wastes and side streams, including lignocellulosic biomass hydrolysates, by-products of biodiesel production, chitin-rich by-products, cheese whey permeate, fungal biomass hydrolysates and microalgal biomass hydrolysates.

At present, SCO from *C. oleaginosus* fermentations has predominantly been used in cosmetic, biofuel and food industries due to its specific product characteristics, such as prevalent long-chain unsaturated fatty acids in the fatty acid profile and the presence of value-adding compounds such as squalene. The continuous advancements in SCO production from *C. oleaginosus*, coupled with its diverse applications, underscore the ongoing progress toward the sustainable sourcing of various oleochemicals.

## Figures and Tables

**Table 1:** Comparison of *C. oleaginosus* with other oleaginous yeasts.

Microorganism	Typical Lipid Content (% DCW)	Notable Advantages	Reference
*Cutaneotrichosporon oleaginosus*	50–85%	Broad substrate tolerance; high lipid yield	Masri et al. (2019) [27], Koruyucu et al. (2023) [28]
*Yarrowia lipolytica*	20–40%	GRAS status; advanced genetic tools	Beopoulos et al. (2009) [29], Ledesma-Amaro et al. (2016) [30]
*Lipomyces starkey*	>70%	Tolerant to high sugar concentrations; high lipid yield	Takaku et al. (2020) [31], Jacob et al. (2023) [32]
*Rhodosporidium toruloides*	>65%	Co-production of carotenoids	Ye et al. (2021) [33]
*Rhodosporidium glutinis*	50–60%	Co-production of carotenoids	Dai et al. (2007) [34]
*Trichosporon fermentans*	>60%	Can tolerate and grow under a variety of stress conditions	Sun et al. (2021) [35], Zhu et al. (2008) [36]

**Table 2:** Summary of the genetic elements employed in the reported genetic engineering approaches with *C. oleaginosus*. Gene acronyms are adapted and standardized. The source organism of the respective gene of interest is indicated in brackets. The whole table was adapted after Stellner (2025) [52]. ACC: Acetyl-CoA carboxylase; ACL: ATP-citrate lyase; ACS: Acetyl-CoA synthetase; ALD: Acetaldehyde dehydrogenase; APH: Aminoglykoside 3′-phosphotransgerase; CFL: Cell flocculin; D12FAD: Δ12-fatty acid desaturase; D9ELO: Δ9-elongase; D9FAD: Δ9-fatty acid desaturase; HMGS: Hydroxymethylglutaryl-CoA synthase; HPH: Hygromycin B phosphotransferase; LAI: Linoleic acid isomerase; NAT: Nourseothricin acetyltransferase; PDAT: Phospholipid–diacylglycerol acyltransferase; PDC: Pyruvate decarboxylase; PDR: Pleiotropic drug resistance; TS: Threonine synthase; URA5: Orotate phosphoribosyl transferase.

Goal	Genes of Interest/Resistance Genes	Transformation Method	Reference
Overexpression; flocculation of cells and facilitated harvesting	CFL1 (*Cryptococcus neoformans*)	Electroporation-assisted random plasmid integration	Donzella et al. (2022) [87]
	HPH (optimized synthetic gene)		
Overexpression; increased TAG production capacity under non-limiting conditions	PDC1 (*Saccharomyces cerevisiae*), ALD6 (*Saccharomyces cerevisiae*), ACS2 (*Saccharomyces cerevisiae*), PDAT (*Rhizopus oryzae*), all codon-optimized (*Ustilago maydis*)	Electroporation-assisted random plasmid integration	Koivuranta et al. (2018) [86]
	HPH (*Escherichia coli*), PDR4 (*Saccharomyces cerevisiae*), APH (*Escherichia coli*)		
Overexpression; increased PUFA content in SCO	D9ELO (*Isochrysis galbana*), D12FAD (*Fusarium moniliforme*), LAI (*Propionibacterium acnes*), all codon-optimized (*C. oleaginosus*)	*Agrobacteria*-mediated random plasmid integration	Görner et al. (2016) [88]
	HPH (*Streptomyces hygroscopicus*)		
Overexpression, gene knockout, promoter replacement: variation in fatty acid saturation in the SCO	D9FAD, D12FAD (*C. oleaginosus*)	Electroporation-assisted, CRISPR-Cas-mediated targeted integration of repair dsDNA in spheroblasts	Shaigani et al. (2023) [84]
	URA5 (*C. oleaginosus*)		
Overexpression; increased TAG production capacity under non-limiting conditions	ACL1, ACC, TS, HMGS (*C. oleaginosus*)	Electroporation-assisted random integration of plasmids (gene of interest and resistance separate) in electrocompetent cells (wild type, Δ9 and Δ12)	Duman-Özdamar et al. (2025) [85]
	NAT		
Overexpression; screening of different resistances and endogenous promoters	-	*Agrobacteria*-mediated random plasmid integration	Stellner et al. (2023) [83]
	HPH (*Streptomyces hygroscopicus*), NAT (*Streptomyces noursei*), APH (bacterial transposon)		

**Table 3:** Alternative substrates as carbon sources for *C. oleaginosus* fermentation and SCO production.

Alternative Substrate	Carbon Sources	Lipid Content (% Lipid per Biomass)	Lipid Titre (g/L)	Fermentation Scale (L)	Fermentation Mode	Reference
Cardoon-stalk hydrolysate	Glucose, xylose, arabinose, galactose and lignin	48.8	7.1	2	Batch and fed-batch	Caporusso et al. (2021) [94]
Lignocellulosic hydrolysate	Xylose, glucose, mannose and acetic acid	75.5	42.1	0.25	Batch and fed-batch	Rerop et al. (2023) [44]
Corn-stover hydrolysate	Glucose, xylose and lignin	64.6	43.2	1	Fed-batch	Wang et al. (2022) [95]
Hydrolyzed herbal extract residues	Glucose, xylose, arabinose, galactose, mannose and acetic acid	40.7	8.5	0.25	Batch	Zhang et al. (2022) [96]
Microalgae biomass hydrolysate	Glucose, mannose, galactose, rhamnose, fucose and ribose	35.0	3.6	0.25	Batch	Younes et al. (2020) [92]
By-products of biodiesel production process	Glycerol	32.0	16	0.1	Batch	Pham et al. (2021) [49]
Hydrolyzed chitin biomass	N-acetylglucosamine, glucosamine and acetic acid	25.0	10.1	0.25	Batch	Tang et al. (2020) [97]
Wheat straw hydrolysate	Glucose, xylose, mannitol and N-acetylglucosamine	65.0	7.5	0.1	Batch	Shaigani et al. (2021) [37]
Cheese whey permeate	Lactose	68.0	38	2	Fed-batch	Donzella et al. (2022) [98]

**Table 4:** Fatty acid profile of SCO produced by *C. oleaginosus* fermentation.

Fatty Acid	Fatty Acid Content Range (%)
C18:1	43–57
C16:0	16–33
C18:0	10–14
C18:2	5–9
C14:0	<1
C16:1	<1
C22:6	<1
C18:3	<1
C22:1	<1
C20:1	<1
C24:0	<0.1
C24:1	<0.1
C20:0	<0.1

## Data Availability

No new data were created or analyzed in this study. Data sharing is not applicable to this article.
